# Role of Atg8 in the regulation of vacuolar membrane invagination

**DOI:** 10.1038/s41598-019-51254-1

**Published:** 2019-10-15

**Authors:** Ayane Ishii, Kazuo Kurokawa, Miyuu Hotta, Suzuka Yoshizaki, Maki Kurita, Aya Koyama, Akihiko Nakano, Yoko Kimura

**Affiliations:** 1 Graduate School of Integrated Science and Technology, Shizuoka University, Shizuoka, 422-8529 Japan; 2 Live Cell Super-Resolution Imaging Research Team, RIKEN Center for Advanced Photonics, Wako, Saitama 351-0198 Japan; 3 Department of Agriculture, Shizuoka University, Shizuoka, 422-8529 Japan

**Keywords:** Lysosomes, Eukaryote

## Abstract

Cellular heat stress can cause damage, and significant changes, to a variety of cellular structures. When exposed to chronically high temperatures, yeast cells invaginate vacuolar membranes. In this study, we found that the expression of Atg8, an essential autophagy factor, is induced after chronic heat stress. In addition, without Atg8, vacuolar invaginations are induced conspicuously, beginning earlier and invaginating vacuoles more frequently after heat stress. Our results indicate that Atg8’s invagination-suppressing functions do not require Atg8 lipidation, in contrast with autophagy, which requires Atg8 lipidation. Genetic analyses of *vps24* and *vps23* further suggest that full ESCRT machinery is necessary to form vacuolar invaginations irrespective of Atg8. In contrast, through a combined mutation with the vacuole BAR domain protein Ivy1, vacuoles show constitutively enhanced invaginated structures. Finally, we found that the *atg8*Δ*ivy1*Δ mutant is sensitive against agents targeting functions of the vacuole and/or plasma membrane (cell wall). Collectively, our findings revealed that Atg8 maintains vacuolar membrane homeostasis in an autophagy-independent function by coordinating with other cellular factors.

## Introduction

Environmental changes and stress can damage organisms and the cells that constitute them. Heat stress on organisms is likely to increase because of the predicted progress of global warming. In general, when cells are exposed to higher temperatures, a number of events are initiated to mitigate the toxic effects and to accordingly adjust various biological processes^1^. For example, during times of heat stress, the activities of protein quality control systems, such as molecular chaperones and protein degradation machinery are enhanced^2^. In addition, heat stress affects cell wall remodelling, which is initiated by activated cell wall stress pathways, including oxidative stress defences, changes in transport systems, membrane fluidity, and energy metabolism^2,3^.

Although responses to heat stress commonly occur at elevated temperatures, yeast cells acquire thermotolerance in different ways depending on the temperature and duration of exposure. During acute and lethal high temperatures, such as 50 °C for 10–20 min, the expression of the molecular chaperone Hsp104 is required for survival. However, a *UBI4* mutant that encodes polyubiquitin, a fusion protein of five ubiquitin copies, can survive^4,5^. In contrast, *hsp104*Δ, but not *ubi4*Δ, mutants can survive chronic heat stress from sub-lethal elevated temperatures, such as at 38.5 °C–41 °C for hours to a day. These conditions may be similar to environmental changes typically experienced on a hot summer day. These findings indicate that many copies of ubiquitins, but not Hsp104, are required for survival under conditions of chronic heat stress.

The reasons for varying requirements for different types of heat stresses are not well understood; however, at lethal temperatures, protein unfolding damage is so severe that the disaggregation of partially-unfolded proteins by Hsp104 may be the most important factor for the rescue of the cell^6,7^. In contrast, under the conditions of sub-lethal and chronic heat stress conditions, the protein-folding damage may not be severe enough to cause cell death. Instead, the cellular systems may be remodelled or reconstructed using ubiquitins to adapt to such heat stress for long-term survival. Evidence suggests that K63-ubiquitination, but not K48-linked ubiquitination, is required for cellular survival^8^. This indicates that processes involving K63-ubiquitination, such as endocytosis and endosomal sorting toward the vacuole, are important for survival^9^. However, what physiological changes or consequences occur in the cell and what cellular changes lead to a heat-tolerant state have not been fully investigated with respect to chronic heat stress.

The vacuole is an extremely dynamic organelle whose morphology changes in response to various stimuli and stresses^10^. Vacuoles fuse during stationary and starvation phases, or under hypotonic conditions, whereas they fission under hypertonic conditions. Vacuolar invaginations may also occur in response to varying stimuli and stresses, with high negative curvature formations of vacuolar membranes induced by heat stress^11,12^. In a previous study, we reported that vacuolar membrane invaginations are enhanced after chronic heat stress^13^. In addition, we showed that invagination formation is impaired in mutants of endosome sorting components and with small invaginations in the *ubi4*Δ mutant. As several plasma membranes proteins degrade more rapidly after heat stress, and with more multivesicular bodies (MVBs) being expected to be delivered and fused with vacuoles under such conditions, the vacuolar invaginations may represent a cellular strategy for coping with increased vacuolar membranes without a massive increase in vacuole volume.

In studies of artificial giant vesicles (GVs), vesicle membrane invaginations are intimately linked with lipid domain formation in the membrane^14,15^. Domains, which are formed by the phase separations of membrane lipid components, cause various changes in vesicle shape^15^. With respect to the yeast vacuole membrane, lipid domains, that segregate vacuole membrane proteins seem to be created in the stationary phase^16–18^. Moreover, localisation analyses of Vph1-GFP have suggested that lipid domains, which appear to correspond with the Vph1-deficient membrane areas, are created in response to several stresses, including nutrient deprivation, translation inhibition, weak acid change and heat stress^13,16^. In addition, the events involved in vesicular trafficking to vacuoles in yeast play a critical role in domain formation on the vacuolar membrane^16^.

Atg8, a member of the ubiquitin-like family of proteins, was initially identified as one of the core elements in autophagic events^19^. During autophagy, Atg8 binds to autophagic receptors and recruits cargo proteins for degradation in the growing autophagosomes^20,21^. Short sequence motifs, termed as Atg8-family-interacting motifs (AIMs), or LC3-interacting regions (LIRs), are responsible for this binding. In addition, Atg8 is conjugated to lipid phosphatidylethanolamine (PE) through a ubiquitin-like conjugative reaction to form an Atg8–PE complex anchored to the membrane^22^. This PE-conjugated Atg8 has been shown to have membrane-tethering and -hemifusion activity *in vitro*, reflecting its *in vivo* activity of autophagosome formation^23^. In addition to participating in autophagy, Atg8 has autophagy-independent functions, including those in vesicular transport, resistance to oxidative stress, vacuolar fusion, and the formation of lipid bodies^24–27^.

In this study, we observed that accelerated invaginations of the vacuole membrane occur after heat stress in *atg8*Δ cells. From the series of results, we showed that Atg8 plays an important role in vacuolar membrane homeostasis in an autophagy-independent function by coordinating with other cellular factors.

## Results

### Accelerated invagination of vacuoles in *atg8*Δ cells after chronic heat stress

**Figure 1:**
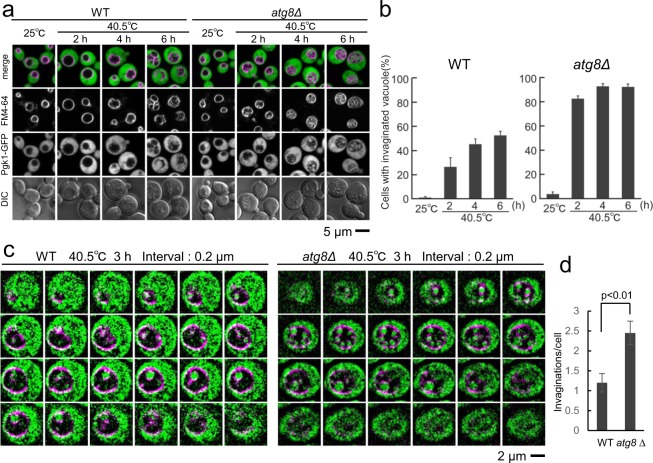
Greater acceleration of invaginations in the vacuolar membranes of *atg8*Δ after chronic heat stress. (**a**) GFP and FM4-64 fluorescence of wild-type and *atg8*Δ cells, expressing PGK1-GFP at 25 °C and 40.5 °C for 2, 4 and 6 h. Scale bar, 5 μm. (**b**) Quantification of (**a**). Cells with invaginated vacuoles were counted. The mean values of the ratio and standard errors (SE) are shown. Statistical significance: p = 0.0045, 0.0013 and 0.0014 by both-sided t-test for all the pairs between wild-type and *atg8*Δ cells at 2, 4 and 6 h, respectively. (**c**) Z-stack analysis of invaginated structures of vacuolar membranes of wild-type and *atg8*Δ cells after 3 h at 40.5 °C. The images were taken by SCLIM. Scale bar, 2 μm. (**d**) Quantification of number of invaginations per cell in (**c**). Invaginations were counted using the serial sections of vacuoles taken by SCLIM, and 20 cells were counted for each. The mean, SE and p-values for both-sided t-test are shown.

Our previous study showed that vacuolar invagination occurs during chronic heat stress without Atg1 or Atg9, indicating that autophagic events are not involved in this process^13^. Although Atg8 is an essential member of the core autophagy machinery, it has autophagy-independent functions^24–27^. In this study, we examined the vacuole morphology of *atg8*Δ mutants after chronic heat stress. We used cytoplasmic PGK1-GFP-expressing cells because vacuolar invaginations are easily detected by GFP fluorescence in the cytosol, and vacuole membranes were stained with FM4-64. The *atg8*Δ mutants showed a normal round vacuolar morphology at 25 °C, indistinguishable from that of wild-type cells (Fig. 1a). After the temperature shifted to 40.5 °C, we observed a surprisingly drastic change in vacuole morphology: massive vacuolar invaginations in the *atg8*Δ mutants compared to that of the wild-type. Time-course observation of the vacuoles further showed that vacuolar membrane invagination occurred earlier after the temperature shift, and more cells had vacuole invagination(s) in the *atg8*Δ mutants than in wild-type cells (Fig. 1b). Using super-resolution confocal live imaging microscopy (SCLIM), additional three-dimensional observation of these cells after 3 h at 40.5 °C showed that more invaginated structures from the vacuolar membrane were created in the *atg8*Δ mutants than in wild-type cells, and confirmed that invaginated structures were continuous with the cytosol around the vacuole (Fig. 1c,d, Videos 1 and 2). It should be mentioned that there was a slight difference in the appearance of vacuoles between wild-type and *atg8*Δ cells after heat stress; some of the vacuolar membranes were rugged in wild-type cells, however, this was rare in *atg8*Δ mutants (Fig. 1a). This greater acceleration of the invagination of *atg8*Δ cells after heat stress was also observed in different strains (Fig. S1a). In addition, such enhanced vacuolar membrane invagination was not observed in the mutant of Atg12, which encodes another ubiquitin-like protein that plays an essential role in autophagy (Fig. S1b). Collectively, these results suggested that Atg8 has a function in suppressing excess vacuolar invaginations after chronic heat stress.

### Heat-inducible expression of Atg8

**Figure 2:**
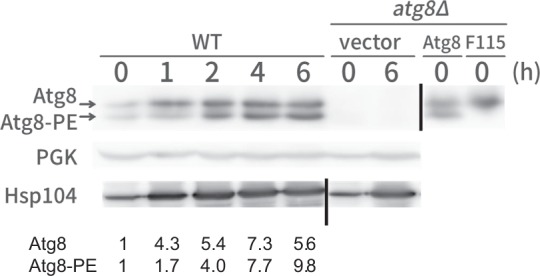
Heat-inducible expression of Atg8. Western blotting analysis of wild-type cells and *atg8*Δ cells after the indicated times at 40.5 °C. Upper, anti-Atg8 immunoblot, PE-form and non-lipidated forms are indicated by arrows. A blot of *atg8*Δ cells expressing Atg8 and non-lipidated form of Atg8-F115 were shown to indicate the positions of Atg8 and Atg8-PE. Middle, anti-PGK1 immunoblot, a control for protein loading and lower, anti-Hsp104 immunoblot, a control for heat shock induction. Lines indicate results are from different blots.

To understand how Atg8 is involved in chronic heat stress, we next examined Atg8 expression levels. The promoter region of the *ATG8* gene contains two STREs (stress responsive elements) in which the heterodimer transcription factor Msn2/Msn4 binds to activate transcription in response to different stresses^28,29^. This suggests a possibility that Atg8 expression is induced after heat stress. To investigate this issue, we performed western blotting using an anti-Atg8 antibody that could detect both PE-conjugated and unconjugated forms of the protein (Figs 2 and S2)^30,31^. As expected, we observed that protein levels of both Atg8 forms increased after heat stress. The amount of the unconjugated form of Atg8 increased precedingly, followed by PE-conjugated form of Atg8. These results suggested that more Atg8 may be used during chronic heat stress.

Although increased expression of Atg8 appeared to suppress enhanced invagination of vacuolar membrane after heat stress, its overexpression in wild-type, which produced mainly PE-unconjugated form of Atg8, did not suppress the invagination frequency after heat stress; vacuolar invaginations were similarly observed in cells overexpressing Atg8 as in control cells (Fig. S3a,b). This suggests that a certain level of Atg8 is required for the suppression of excess invagination, but excess Atg8 does not suppress further invagination.

In addition, we examined whether lipid droplets (LDs) were involved in the vacuolar invaginations. It has been demonstrated that LDs, vacuoles and Atg8 are related. In lipophagy, LDs are delivered to the vacuoles through autophagy^32,33^. In the stationary phase, LDs reportedly show a small decrease and small increase in *atg8*Δ and Atg8-overexpressing wild-type cells, respectively^27^. Moreover, we previously showed that LDs considerably increase after chronic heat stress^13^. Co-staining of LDs and vacuolar membrane with BODIPY493/503 and FM4-64, respectively, showed that some LDs but not many of the invaginations were colocalised with the invagination in *atg8*Δ cells (Fig. S4a). Neither deletion of *atg8*Δ nor overexpression of Atg8 significantly affected the number of LD compared with the control after 4 h at 40.5 °C (Fig. S4b). Therefore, these results suggested that LDs were not directly involved in invagination formation after chronic heat stress, which we observed in this study. However, considering the existence of LDs close to the vacuoles after chronic heat stress and the limitation of detection for very small LDs, involvement of LDs in the vacuolar invaginations is not completely deniable.

### PE-conjugation is dispensable for Atg8 to suppress vacuolar invagination

**Figure 3:**
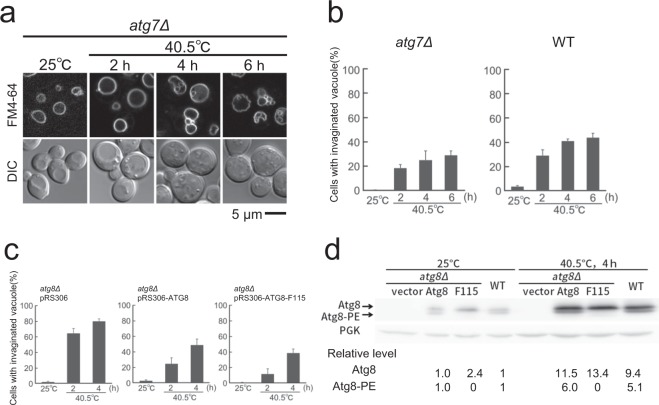
Lipidation-independent function of Atg8 for suppressing vacuole invagination after heat stress. (**a**) FM4-64 staining of vacuolar morphology of *atg7*Δ mutants at 25 °C and after the indicated times at 40.5 °C. Because FM4-64 fluorescence was fainter in cells at 25 °C than in cells after heat stress, the contrast of the images of cells at 25 °C was enhanced. Images with the same contrasts at 25 °C and 40.5 °C are shown in Fig. S5a. (**b**) Quantification of invaginated structures of vacuolar membrane in (A). The mean values of the ratio and standard errors (SE) are shown. (**c**) Quantification of invaginated structures of vacuolar membrane in *atg8*Δ cells (pRS306) and *atg8*Δ cells expressing Atg8 or Atg8-F115. SEs are shown. Statistical significance: p = 0.01 and 0.006 for both-sided t-test for *atg8*Δ cells (pRS306) vs. *atg8*Δ cells (pRS306-ATG8-F115) both at 2 and 4 h, respectively at 40.5 °C. Statistical significance was not observed (p > 0.05) for *atg8*Δ (pRS306-ATG8) vs. *atg8*Δ (Atg8-F115) cells both at 2 and 4 h at 40.5 °C, respectively. (**d**) Expression of Atg8 and Atg8-F115. Western blot analysis was performed using cells in (C) and wild-type cells at 25 °C and at 40.5 °C for 4 h, with anti-Atg8 and anti-PGK1 antibodies. Quantification was shown at the bottom. Uncropped western blotting results are presented in Fig. S5b,c.

PE-conjugation to Atg8 is necessary for the autophagy process; however, it has been shown that it is not required for some of the autophagy-independent functions of Atg8^25–27^. The PE-conjugation of Atg8 occurs as a series of events in a ubiquitination-like reaction^22^. The cysteine protease Atg4 first hydrolyses Atg8’s C-terminal residue, exposing a Gly residue at the C-terminus. Then the E1-like enzyme Atg7 activates Atg8, forming an adenylated intermediate. The Atg8 intermediate is then transferred to the E2-like enzyme Atg3, and PE is attached to Atg8. To investigate whether invagination suppression by Atg8 is a PE-lipidation dependent process, we first examined vacuolar membrane invagination in *atg7* mutant in which PE-conjugation to Atg8 does not occur. As shown in Fig. 3a,b, excessive invagination was not observed in *atg7* mutant after chronic heat stress. Similar results were obtained in both the *atg3*Δ and *atg4*Δ mutants (Fig. S1). There results suggest that suppression of excess vacuolar invagination by Atg8 does not require PE.

To prove that PE-lipidation of Atg8 is not required for this process, we next introduced *ATG8* or the *atg8* gene, which encodes Atg8-F115 lacking the critical Gly residue for lipidation, into *atg8*Δ mutants^27^. We observed that lipidation-defective Atg8-F115 expression suppressed excess invagination in vacuolar membranes of *atg8*Δ cells after heat stress (Fig. 3c). We confirmed that Atg8’s lipidated form was not expressed in cells expressing Atg8-F115 (Figs 3d and S5). These results indicated that Atg8’s suppression of vacuolar invagination was an autophagy-independent process, and that Atg8’s lipidation was not required for the observed suppression.

### Relationship between Atg8 and other factors

**Figure 4:**
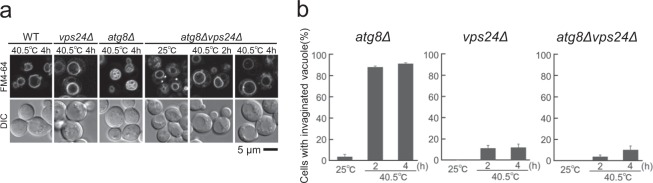
Relationship of Atg8 with ESCRT factors. (**a**) Vacuolar morphologies of wild-type, *vps24*Δ, *atg8*Δ, and *atg8*Δ*vps24*Δ cells at 25 °C and 40.5 °C. Because FM4-64 fluorescence was fainter in cells at 25 °C than in cells after heat stress, the contrast of images of cells at 25 °C was enhanced. Images with the same contrasts at 25 °C and 40.5 °C are shown in Fig. S6b. (**b**) Quantification of cells with invaginated vacuoles. Cells with invaginated vacuole structures were counted among a total of 50 cells in each experiment, and the three independent experiments were performed. The mean values of the ratio and standard errors (SE) are shown. Statistical significance was not observed (p > 0.05) for *vps24*Δ cells vs. *atg8*Δ *vps24*Δ cells both at 2 and 4 h at 40.5 °C, respectively.

To investigate the nature of the accelerated invaginations observed in *atg8*Δ cells, we next examined the vacuolar morphology of *atg8*Δ double mutants with a different gene mutation. The ESCRT apparatus, comprised of four complexes (ESCRT-0, I, II, and III), is responsible for sorting ubiquitinated membrane proteins to the lysosome/vacuole for degradation^34^. In a previous study, we showed that vacuolar membrane invaginations are not significantly produced after chronic heat stress in Vps23 and Vps24 mutants, which are components of ESCRT I and III, respectively^13^. Therefore, we first examined the vacuoles in the *atg8*Δ*vps24*Δ mutant (Fig. 4a,b). Vacuolar invaginations were severely impaired in the double mutants after heat stress, which resembled the phenotype of *vps24*Δ^13^. Similar results were obtained for the *atg8*Δ*vps23*Δ mutant (Fig. S6a). These results indicated that Vps24 or Vps23 were epistatic to Atg8. As these mutants were sensitive to chronic heat stress, we investigated mutant vacuoles only up to 4 h of heat treatment.

**Figure 5:**
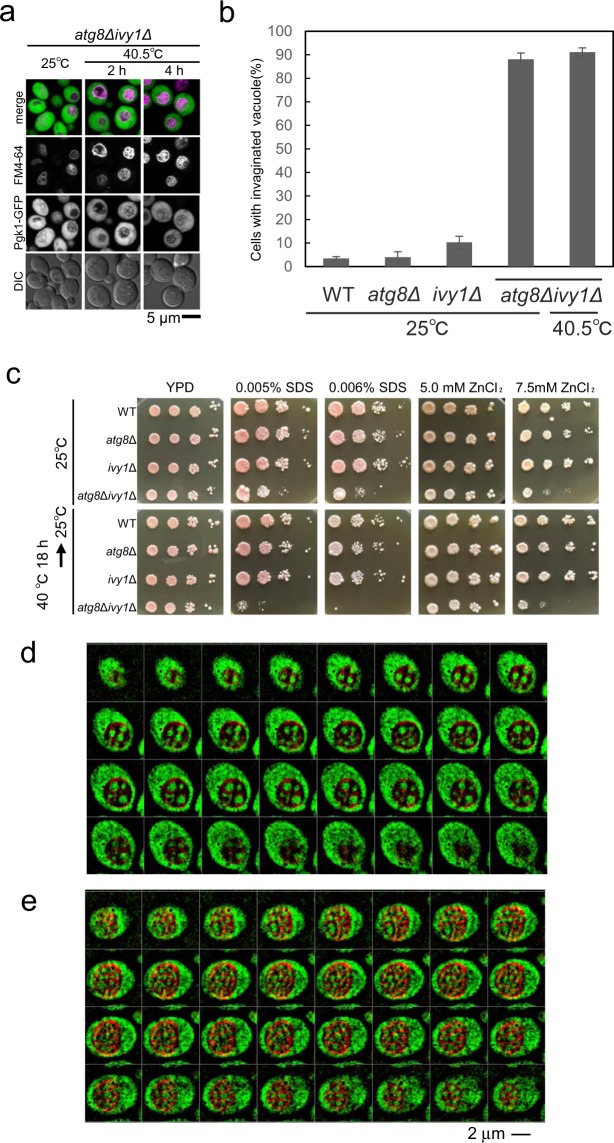
Relationship of Atg8 with Ivy1. (**a**) Enhanced vacuolar invaginations of *atg8*Δ*ivy1*Δ cells expressing Pgk1-GFP at 25 °C and 40.5 °C for 2 and 4 h. (**b**) Quantification of vacuolar invaginations of wild-type, *atg8*Δ, *ivy1*Δ, and *atg8*Δ*ivy1*Δ cells at 25 °C, and *atg8*Δ*ivy1*Δ cells at 40.5 °C for 4 h. The mean values of the ratio and standard errors (SE) are shown. (**c**) Sensitivities against SDS and ZnCl_2_. Serial 10-fold dilutions of indicated strains grown at log phase were spotted on YPAD, YPAD + 0.005% SDS, YPD + 0.006% SDS, YPAD + 5.0 mM ZnCl_2_, and YPAD + 7.5 mM ZnCl_2_ plates. Cells were placed at 25 °C or at 40 °C for 18 h followed by the incubation at 25 °C. Cells were incubated at 25 °C for 3 days for YPAD, 6 days for SDS-containing cells, and 8 days for ZnCl_2_ containing plates. (**d**,**e**) Z-stack analysis (0.1 μm spacing) of FM4-64 treated *atg8*Δ*ivy1*Δ mutants expressing PGK1-GFP with (**e**) or without 0.006% SDS (**d**) for 3 h at 25 °C. Merged images of GFP and FM4-64 fluorescence are shown. Scale bar, 2 μm. The images were taken by SCLIM.

Ivy1 is a vacuole-localised protein with an Inverted-BAR (I-BAR) domain^12,35^^,^. It is localised in vacuolar membranes after stationary phase and heat stress at domains that exhibit negative curvature with respect to the cytosolic face, in the vacuolar membrane; however, *ivy1*Δ mutants exhibit normal vacuolar invaginations after heat stress^12,13,16^^,^. In the *ivy1*Δ mutant, Atg8 was expressed at a level similar to that in wild-type both at 25 °C and 40.5 °C (Fig. S7). Double mutants harbouring *ivy1*Δ and *vma16*Δ, a mutation in the gene encoding a vacuolar ATPase subunit, have been reported to have vacuoles with enlarged and invaginated vacuolar surfaces that resemble the vacuolar morphology of *atg8*Δ cells after heat stress^12^. We therefore examined these *atg8*Δ*ivy1*Δ double mutants to evaluate the relationship between Atg8 and Ivy1. Interestingly, we observed vacuoles with many membrane structures, even at the normal temperature of 25 °C, which resembled the vacuoles in *atg8*Δ cells after heat stress (Fig. 5a,b). Z-stack analysis showed that the vacuoles had constitutive cytoplasmic invaginations (Fig. 5d). Introduction of a plasmid expressing either Atg8 or Ivy1 to the *atg8*Δ*ivy1*Δ cells cancelled the abnormal invaginated vacuole structures (Fig. S8a). These results suggest that either Ivy1 or Atg8 is required to suppress vacuolar membrane invaginations at the normal temperature, whereas Atg8 is required after chronic heat stress.

The *atg8*Δ*ivy1*Δ mutant grew slightly more slowly than the wild-type, *atg8*Δ and *ivy1*Δ cells at 25 °C. Although the *atg8*Δ*ivy1*Δ mutants grew at higher temperatures, its growth was reduced at high concentrations of ZnCl_2_, which would affect vacuolar activity (Fig. 5c)^36^. In addition, *atg8*Δ*ivy1*Δ mutant exhibited a severe growth defect when they were plated on a medium containing SDS, which perturbates plasma membrane and cell walls (Fig. 5c)^37^. This defect was pronounced when the cells were plated on ZnCl_2_ or SDS-containing media and were heat-stressed for 18 h before incubation at 25 °C. Introducing a plasmid expressing Ivy1 or Atg8 to the *ivy1*Δ*atg8*Δ mutants rescued these sensitivities (Fig. S8b). Moreover, we observed that vacuole invaginations became further intricated by SDS treatment in the mutant at 25 °C (Fig. 5e). Collectively, these results support the importance of suppressing excess vacuolar invaginations for cell growth and the maintenance of vacuolar membrane homeostasis.

**Figure 6:**
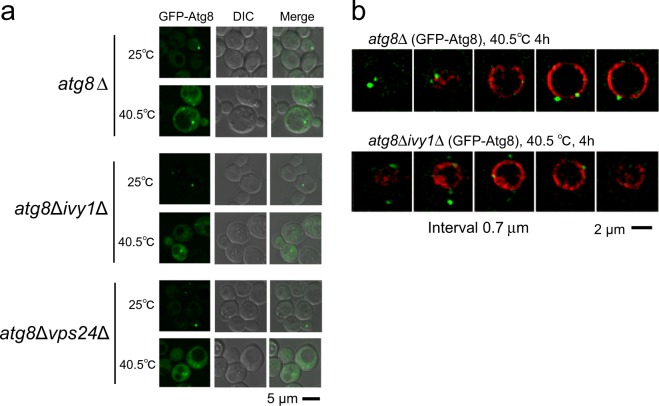
Localisation of GFP-Atg8. (**a**) Localisation of GFP-Atg8 in cells incubated at 25 °C and at 40.5 °C for 4 h: a plasmid expressing GFP-Atg8 under the control of *ATG8* promoter was expressed in *atg8*Δ, *atg8*Δ*ivy1*Δ, and *atg8*Δ*vps24*Δ cells. (**b**) Z-stack analysis of GFP-Atg8 dots observed in control, *atg8*Δ, and *atg8*Δ*ivy1*Δ cells at 40.5 °C for 4 h. Merged images of GFP and FM4-64 fluorescence are shown. The images were taken by SCLIM, and images at 0.7 μm intervals are shown. Scale bar, 2 μm.

To understand how Atg8 or Ivy1 are related to vacuole invaginations and heat stress, we investigated the localisation of GFP-Atg8 and Ivy1-GFP. To assess the localisation of Atg8, a plasmid expressing GFP-Atg8 under the control of *ATG8* promoter was introduced into *atg8*Δ, *atg8*Δ*vps24*Δ or *atg8*Δ*ivy1*Δ cells (Fig. 6). The GFP-Atg8 fluorescence intensity varied with cells, possibly because GFP-Atg8 was exogenously expressed. We still observed that its cytoplasmic fluorescence increased overall after 4 h at 40.5 °C (Fig. 6a). In some cells, GFP-Atg8 fluorescence was detected in the form of punctate structures around the vacuole membranes both at 25 °C and 40.5 °C. The punctate structure was probably a PAS (pre-autophagosomal structure), where the autophagosome has been proposed to be generated, and it has been reported that PAS exists even under nutrient-rich conditions^38,39^. The GFP-Atg8 punctate structures were further examined with a SCLIM together with FM4-64 fluorescence (Fig. 6b). After the heat stress, the number of the punctate structures in a cell increased in *atg8*Δ and *atg8*Δ*ivy1*Δ cells but not in *atg8*Δ*vps24*Δ; the average numbers among cells harbouring them at 25 °C and 40.5 °C were 1.1 and 1.9 in *atg8*Δ cells, 1.3 and 2.4 in *atg8*Δ*ivy1*Δ cells and 1.7 and 1.8 in *atg8*Δ*vps24*Δ cells. About 10%–20% of cells possessing the punctuate structure(s) contained more than two in a cell after heat stress. These punctate structures were mainly localised on the vacuole membrane or close to the vacuole membrane, and they were observed both at invaginated and un-invaginated areas on the vacuolar membrane. In addition, some of them were localised where FM4-64 fluorescence was not detected, suggesting a possibility that they were localised at the cytosol or at new vacuoles which were not stained with FM4-64. Therefore, although we observed these changes after heat stress, it is unclear how the localisation of Atg8 is related to vacuolar invagination after heat stress at present.

**Figure 7:**
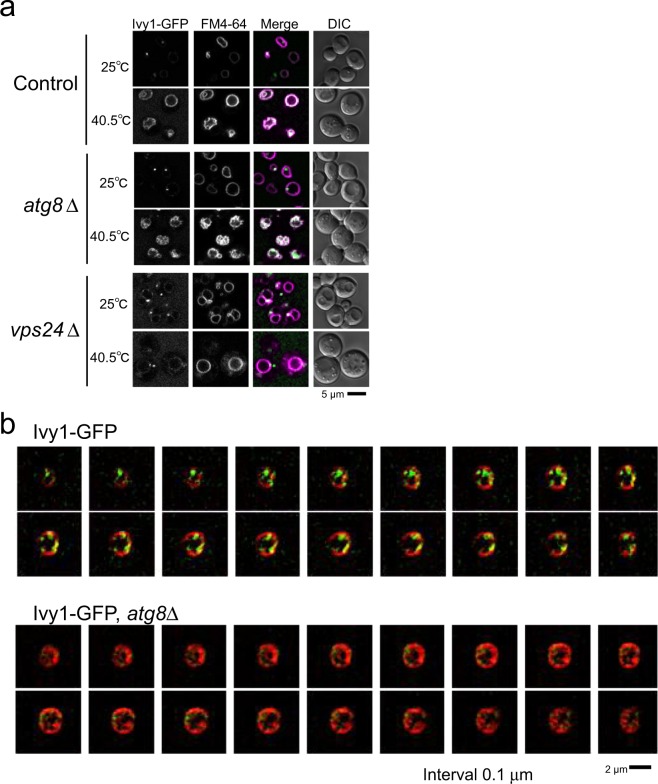
Localisation of Ivy1-GFP. (**a**) Localisation of Ivy1-GFP expressed in control, *atg8*Δ, *vps24*Δ cells incubated at 25 °C and at 40.5 °C for 4 h. Images were taken by SCLIM. (**b**) Z-stack analysis of Ivy1-GFP in control, *atg8*Δ, and *atg8*Δ*ivy1*Δ cells incubated at 40.5 °C for 4 h. Merged images of GFP and FM4-64 fluorescence are shown. The images were taken by SCLIM. Scale bar, 2 μm.

It has been reported that Ivy1-GFP is localised mainly as dots on the vacuolar membrane at the normal temperature, whereas it is distributed at the domains of negative curvature of invaginations of the vacuolar membrane after 1 h at 42 °C^12^. Similar results were noted when we examined the localisation of Ivy1-GFP in the wild-type and *atg8*Δ mutants at 25 °C and 40.5 °C for 4 h (Fig. 7). At 25 °C, GFP fluorescence of Ivy1-GFP was detected mainly as dots on the vacuolar membrane with a very faint fluorescence along with vacuolar membrane (Fig. 7a). After heat stress, Ivy1-GFP distributed and scattered along the vacuolar membrane in control and *atg8*Δ cells as observed by SCLIM (Fig. 7b). Ivy1-GFP localisation was not considerably changed both at 25 °C and 40.5 °C for 4 h in the *vps24*Δ mutant, which does not show obvious invaginations of the vacuolar membrane after heat stress, and Ivy1-GFP were localised mainly as dots on the vacuoles (Fig. 7a). From these results, it was suggested that Ivy1 localisation change after heat stress depends on Vps24 and it may depend on the formation of invagination on the vacuolar membrane.

## Discussion

In this study, we demonstrated that vacuolar membrane invagination is a controlled process, likely to maintain membrane homeostasis, and that Atg8 plays an important role in the process.

Biophysical analyses of artificial tertiary-component GVs show that lipid domain formations, which are caused by the phase separation of different lipids, lead to the formation of various shape in the vesicles^14,15^. Moreover, it has recently been reported that a cycle of quick temperature increases and decreases result in the disappearance and reappearance of micro-scale domains, which are formed in the yeast vacuoles in the stationary phase and disappear and reform at a certain distinct temperature^18^. These results suggest that the lipid domain formed in the vacuole membrane might cause the vacuole invaginations. However, more complex events seem to occur when chronic heat stress is applied to growing cells that are unlikely to have lipid domains on their vacuole membranes at normal temperatures. Heat stress seems to facilitate lipid domain formation on the vacuolar membrane in yeast, as suggested by Vph1-GFP localisation analysis^13,16^. In addition, we showed here that Atg8 is required for the prevention of excess invagination after heat stress. Therefore, several events during chronic heat stress in growing cells, such as the introducing different lipids or proteins to the vacuole membrane or other unidentified events, may antagonise the biophysical changes in existing vacuole membranes caused by temperature increase.

We speculate that the role of Atg8 in vacuolar invaginations may be to regulate the formation of lipid domains although we have not been able to obtain clear results for different Vph1-GFP localisations on the vacuole membrane in *atg8*Δ cell compared to wild-type after heat stress (data not shown). Atg8 may inhibit lipid domain formation of the vacuole membrane or promote merging multiple lipid domains. It has been reported that in the stationary phase, when lipid domains are created, a *atg8*Δ mutant is defective in the formation of lipid domains as suggested by Vph1-GFP localisation^40^. Therefore, the loss of Atg8, which may otherwise lead to the appropriate domain formation in the vacuolar membrane, may result in the aberrant invagination formation after chronic heat stress. Alternatively, Atg8 may regulate either positively or negatively lipid domain formations, depending on the conditions.

Another possibility of Atg8 function is that Atg8 may inhibit the MVB fusion with vacuolar membranes after heat stress, and not incorporate the excess membrane area by fusions with MVB. This speculation is based on the report that Atg8 binds to fusion machinery, such as HOPs, at the fusion of autophagic bodies and the vacuole during autophagy^41^. One more possibility is that Atg8 may promote the release of a portion of the vacuolar membrane from the vacuole besides fission, although such a phenomenon has not been reported in yeast. Our results indicate the *atg8*Δ*ivy1*Δ mutant enhanced growth retardation in the presence of SDS, which perturbs the plasma membrane/cell wall. Thus, an excess vacuolar membrane may be retrieved to the plasma membrane to compensate for the loss of plasma membrane by endocytosis. In this sense, vacuole membranes may have a function as a storage place or buffer for cellular membranes in order to maintain cellular membrane homeostasis.

It has been reported that PE-conjugated Atg8 generates membrane curvature, whereas our analysis showed that Atg8 lacking PE appears to inhibit the formation of vacuolar invaginations^42^. In other words, Atg8 without PE may inhibit curvature formation in the membrane. In this case, Atg8 lacking PE may have an opposite function to Atg8-PE. Alternatively, since Atg8 can bind to other molecules through its AIM, it may exert its function through binding proteins^21^. Recently, Hfl1, a vacuolar membrane protein, was identified as a lipidation-independent Atg8 binding protein^43^. Thus, proteins such as Hfl1 may help mediate the suppression of vacuolar invagination by Atg8. Although the relationship between Atg8 localisation and vacuolar invagination remains unclear at present, further analysis of the localisation studies of Atg8 and related molecules such as Hfl1 may unravel how Atg8 acts on the suppression of the vacuolar invagination.

It should be mentioned that, after heat stress, vacuole fissions of *atg8*Δ cells were suppressed compared to wild-type after heat stress (data not shown). Therefore, vacuolar fission impairment in *atg8*Δ after heat stress may be one reason for the observed accelerated invagination because the excess vacuolar surface area was not cancelled by vacuole fissions. However, vacuole fission defective mutants of *vac14*Δ and those of *vac7*Δ did not show such invaginations of the vacuole membranes after heat stress (data not shown)^10^. Thus, we do not think only vacuole fission impairment by *atg8*Δ after heat stress would cause enhanced vacuolar invagination.

Since the introduction of *atg8*Δ did not change the smooth vacuolar morphologies of the *vps24*Δ or *vps23*Δ mutants, the full ESCRT machinery appears to be required for invaginating vacuolar membranes, irrespective of the presence of Atg8. After heat stress, several plasma membrane proteins are ubiquitinated, transported through endosomes and degraded in the vacuoles, with ESCRT proteins playing critical roles in this sorting process^44^. It may be that, after heat stress, more MVBs fuse with the vacuolar membranes, and more sterols are released from the intraluminal vesicles (ILVs) of MVBs in the vacuole lumen to be delivered to the vacuolar membrane. If so, bud-like invaginations may be formed due to the inflow of membrane components to the vacuole membranes. This inflow may trigger lipid domain formation on the vacuole membrane that Atg8 acts on to suppress.

Furthermore, we have shown that constitutive vacuolar invagination was observed even at the normal temperature in *atg8*Δ*ivy1*Δ mutants. We, therefore, propose that vacuolar membrane invagination is a regulated process in cells, and that factors such as Atg8 and Ivy1 are required to suppress invagination at normal temperatures in order to keep vacuoles in their sphere form. The I-Bar domain, which is present in Ivy1 has been reported to bind to the membrane curvature^45^. Ivy1 binds to phospholipids, along with several vacuole-related factors, including Ypt7 and Vps33^12,35^. The result that Ivy1 localisation did not change in *vps24*Δ mutants after heat stress in this study suggests that Ivy1 might move to the invaginated places after the invaginations are made. Recently, it has been reported that Ivy1 functions as an inhibitor of Fab1, PI-3,5-kinase, and a negative regulator of TORC activation^46,47^. These functions may therefore be related to vacuolar invagination suppression by Atg8. In addition, it has been reported that the vacuoles in the *ivy1*Δ*vma16*Δ mutants also exhibit the membranous structures at normal temperatures, which suggests that Atg8 and Vma16 may share a common function in suppressing invagination in conjunction with Ivy1^12^.

Finally, as yeast is a model organism for various eukaryotes, we believe that a similar vacuolar phenomenon might occur in the cells of diverse organisms after chronic heat stress.

## Methods

### Media, yeast strains and plasmids

Yeast strains were grown in YPAD medium [1% yeast extract, 2% Bacto–Peptone or Hipolypepton (Nihon Seiyaku), 2% glucose, and 0.002% adenine], in synthetic complete medium (SD; 0.67% yeast nitrogen base and 2% glucose supplemented with amino acids), or synthetic casamino medium (SC; 0.67% yeast nitrogen base, 2% glucose, and 0.5% casamino acids; if necessary, tryptophan, uracil or adenine was added). A list of yeast strains is provided in Table S1. W303 strains were used if not indicated.

The integration plasmids, pRS306-ATG8 and pRS306-ATG8-F115 and the centromere plasmid, pRS316-ATG8, were kindly provided by Drs. Y. Sakai and M. Oku^27^. The plasmid pRS316-IVY1 expressing Ivy1 was obtained by cloning IVY1 DNA by PCR using the oligonucleotides AATTGTCGACCCATCTCTGCCGGGGCAATG and AATTGGATCCATAATGAAAAGAGAGTATTA and by using genomic DNA as a template, cutting the PCR product with BamHI and SalI, and ligating it to the BamHI and SalI fragment of pRS316. The plasmid pRS316-GFP-ATG8 that expresses yoEGFP-ATG8 under the control of *ATG8* promoter (−1000 to −1) was created by the Gibson Assembly method^48^. The yoEGFP region was amplified using pFA6a-yoEGFP-SpHis5 (Addgene) as a template.

### Western blotting for detection of Atg8 and Atg8-PE

We prepared whole-cell extracts and performed immunoblot analysis essentially as previously described^49^. Cells (1–3 × 10^7^) were washed with water and suspended in 200 μL of cold ethanol containing 2 mM PMSF. Cells were lysed by agitation with 200 μL of glass beads for 10 min and chilled at −20 °C. Cells were then dried, suspended in sample buffer and heated at 95 °C for 5 min. Western blotting for the detection of Atg8 and Atg8-PE was performed according to the method described by Kirisako *et al*.^30^. Briefly, a 6 M urea containing 14% SDS-PAGE gel was used to separate non-lipidated and lipidated forms of Atg8^30^. Polyclonal rabbit anti-Atg8 antibody (a gift from Dr. Ohsumi) was used to detect both forms of Atg8^31^. For other western blotting experiments, blots were incubated with rabbit anti-Hsp104 antibody (Stressgen) or anti-yeast phosphoglycerate kinase (PGK) antibody (Molecular Probes), followed by horseradish peroxidase (HRP)-conjugated anti-mouse IgG (#NA931V, GE Healthcare): blots were then visualised using a chemiluminescent reagent.

### Microscopy

FM4-64 staining was performed as described previously^50^, and the cells were treated with FM 4-64 just before the temperature shift. To treat FM4-64, a 1.5 mL culture of cells was grown at 25 °C in YPAD medium, followed by centrifugation and suspension in 49 μL of YPAD. To the cells, 1 μL of 2 mM FM4-64 (Molecular Probes) was added at a final concentration of 40 μM and incubated for 20 min at room temperature. The cells were then washed with 1 x PBS and suspended in 2 mL of YPAD, followed by the heat treatment. Cells harbouring a plasmid expressing GFP-Atg8 were grown in SC-Ura medium to log phase, and the YPAD medium was used for the FM4-64 treatment and the following heat-stress treatment. To stain lipid bodies, 4 μl of 1 mg/ml of BODIPY493/503 (Invitrogen) was added to 3 ml of culture for the last 10 min of the heat treatment. After heat treatment, the cells were collected by centrifugation and were put in a heat block before subjecting them to microscopy. Cells were imaged at room temperature using a confocal microscope (LSM700; Carl Zeiss) equipped with a 100× oil objective lens. Images were processed, and the brightness and the contrast were adjusted, using Zen software. For quantifications, cells with invaginated vacuole structures were counted, and at least three independent experiments were performed.

### Fluorescence microscopy by super-resolution confocal live imaging microscopy (SCLIM)

Super-resolution confocal live imaging microscopy (SCLIM) was developed by combining Olympus model IX-71 inverted fluorescence microscope with a UPlanSApo 100 X NA 1.4 oil objective lens (Olympus, Japan), a high-speed and high signal-to-noise ratio spinning-disc confocal scanner (Yokogawa Electric, Japan), a custom-made spectroscopic unit, image intensifiers (Hamamatsu Photonics, Japan) equipped with a custom-made cooling system, magnification lens system for providing 266.7 X final magnification, and three EM-CCD cameras (Hamamatsu Photonics, Japan) for green, red and infrared observation. Image acquisition was executed by a custom-made software (Yokogawa Electric, Japan)^51^. For observation, cells were collected by centrifugation and suspended in SC medium. Cell cultures were plated on a cover glass with concanavalin A and left for 3 min on a heat block at 40.5 °C for heat-treated cells or at room temperature for untreated cells to allow the cells to attach to the cover glass. SC medium was removed by aspiration, and pre-warmed YPAD was added. The cells were imaged by SCLIM on a thermos-controlled stage whose temperature was kept at 40.5 °C for heat-treated cells. Z-stacks were acquired at 0.1-μn spacing by oscillating the objective lens vertically with a custom-made piezo actuator. Z-stack images were processed by deconvolution with Volocity software (Perkin Elmer, MA) using the theoretical point-spread function for spinning-disc confocal microscopy. Deconvolved z-stack images were converted to 3D images with Volocity software.

## Supplementary information

Supplementary info

Video 1. 3D images of the vacuole in a wild-type cell after 3 h at 40.5°C

Video 2. 3D images of the vacuole in an atg8 cell after 3 h at 40.5°C.

## Data Availability

No datasets were generated or analysed during the current study.
