# Supplementary material for: Role of Atg8 in the regulation of vacuolar membrane invagination
Source: Sci Rep. 2019 Oct 15;9:14828. doi: 10.1038/s41598-019-51254-1 (PMC6794316; doi:10.1038/s41598-019-51254-1)
Supplement: Supplementary file 1 — Supplementary info [file 41598_2019_51254_MOESM1_ESM.pdf]

Supplementary Figures and Table

The role of Atg8 in the regulation of vacuolar membrane invagination

Ayane Ishii[a], Kazuo Kurokawa[b], Miyuu Hotta[c], Suzuka Yoshizaki[c], Maki Kurita[a], Aya Koyama[c], Akihiko Nakano[b], and Yoko Kimura[a,c,*]

Graduate School of Integrated Science and Technology, Shizuoka University, Shizuoka, 422-8529, Japan[a]; Live Cell Super-Resolution Imaging Research Team, RIKEN Center for Advanced Photonics, Wako, Saitama 351-0198, Japan[b]; Department of Agriculture, Shizuoka University, Shizuoka, 422-8529, Japan[c]

Figure S1

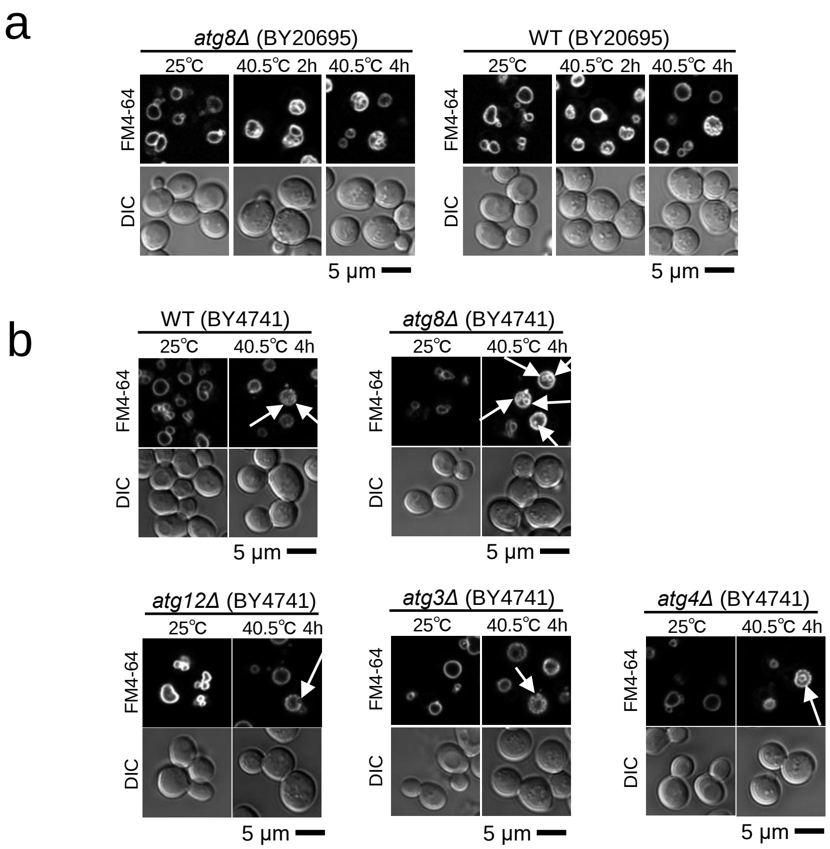

Figure S1. FM4-64 staining.

(a) Strain BY20695 and *atg8*Δ cells.

(b) FM4-64 staining of BY4741, *atg8*Δ, *atg3*Δ, *atg4*Δ, and *atg12*Δ incubated at 40.5° C for 4 h. Arrows indicate vacuolar invaginations

Figure S2

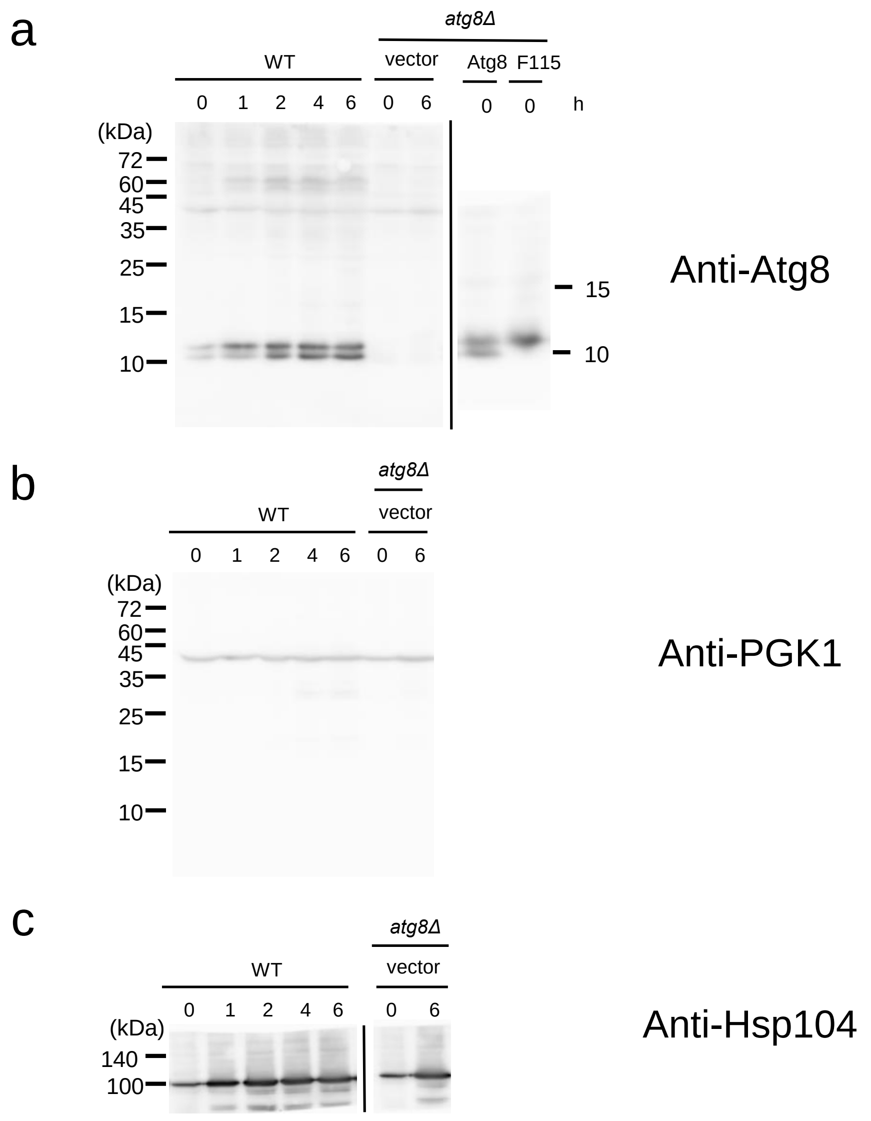

Figure S2. Uncropped images of the blots shown in Fig.2.

(a) Anti-Atg8 antibody.

(b) Anti-PGK1 antibody.

(c) Anti-Hsp104 antibody.

Figure S3

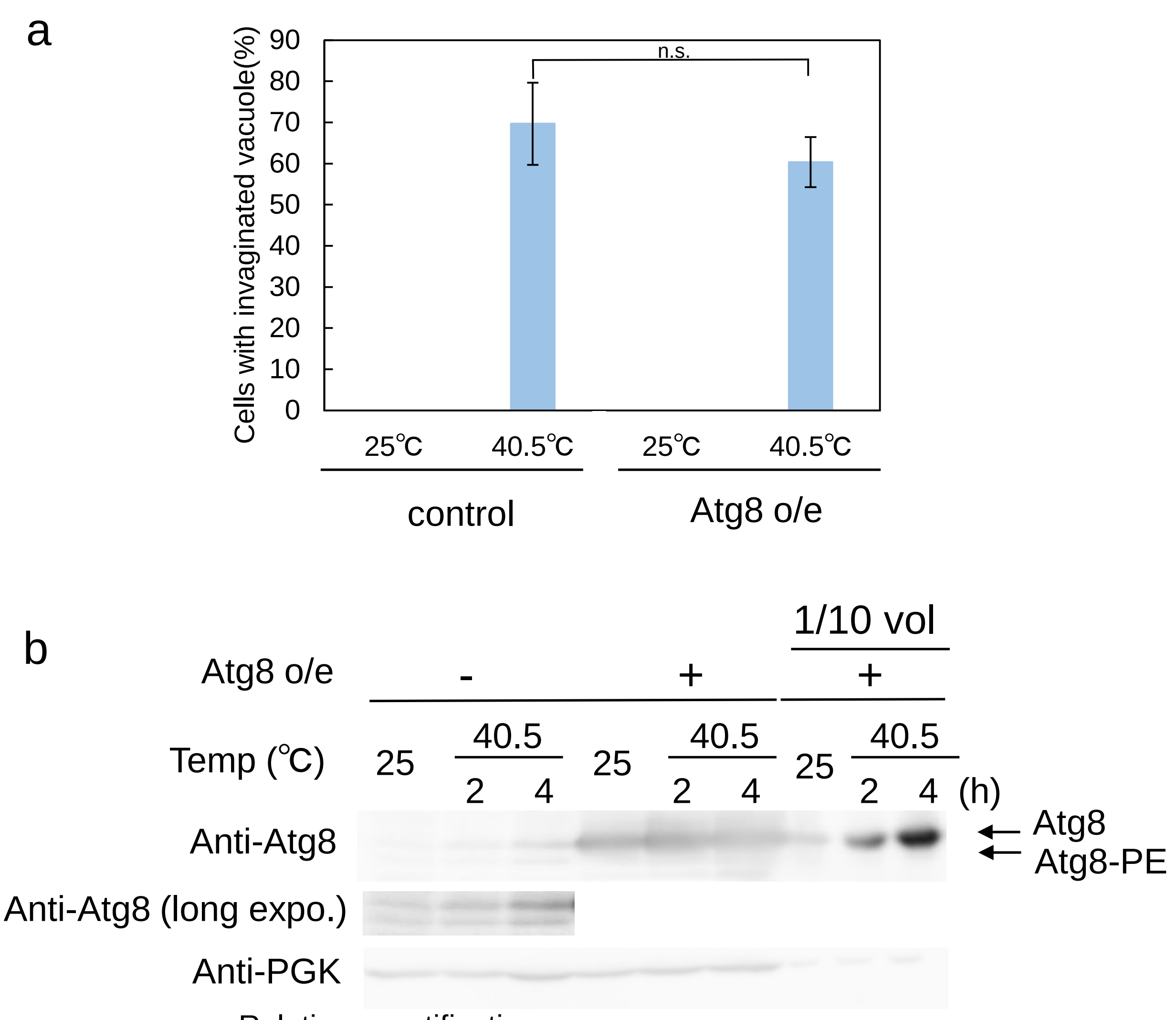

Relative quantifications

Atg8 o/e	-			+		
Temp (℃)	25	40.5		25	40.5	
		2	4		2	4 (h)
Atg8	1	2.4	5.4	83	474	1470
Atg8-PE	1	1.6	3.7	6.5	8.0	7.4

Figure S3. Overexpression of Atg8

(a) Quantifications of vacuolar invaginations of wild-type cells harbouring a control plasmid or a plasmid overexpressing Atg8 at 25 ° C and at 40.5° C for 4 h. Cells were grown in SC-ura media to log phase and heat shocked for 4 h. The mean values of the ratio and standard errors (SE) are shown. $p>0.05$ for t-test.

(b) Western blotting with anti-Atg8 and anti-PGK1. One-tenth vol of lysates of wild-type cells overexpressing Atg8 were also examined. An image with a longer exposure time was added. Relative quantifications were shown at the bottom. Quantifications of cells overexpressing Atg8 were calculated from the results of 1/10 vol of lysates.

Figure S4

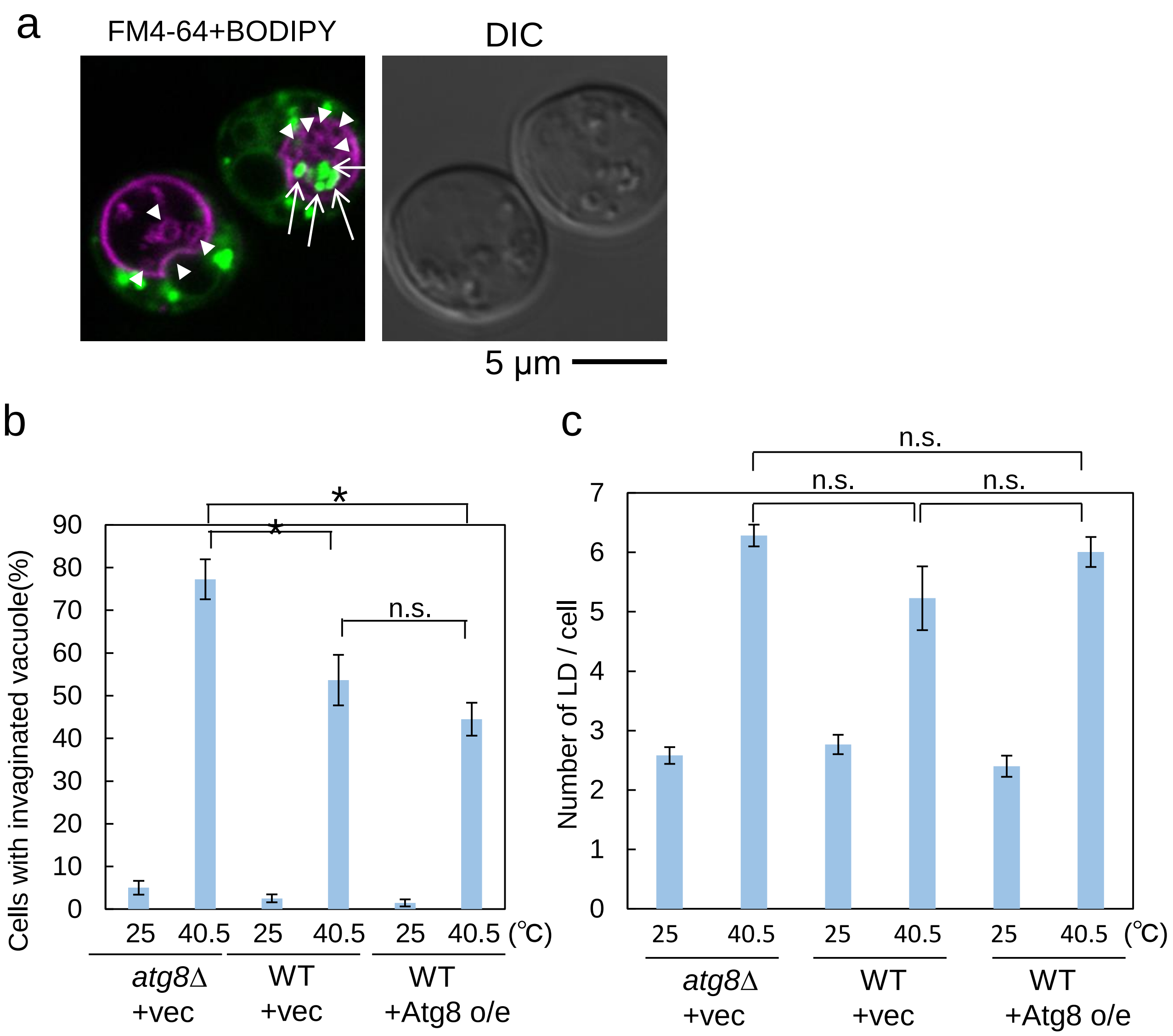

Figure S4. Relationship with LDs

(a) BODIPY493/503 and FM4-64 fluorescence in *atg8*Δ cells incubated at 40.5° C for 4 h. Arrows and arrowheads indicate vacuolar invaginations with LDs and vacuolar invaginations without LDs, respectively.

(b) Quantification of vacuolar invaginations by *atg8*Δ deletion or Atg8 overexpression. Cells harbouring a vector or 2µm-based Atg8-expressing plasmid were grown in SC-Ura media. Cells were centrifuged and suspended in YPD and heat shocked for 4 h. The mean values of the ratio and standard errors (SE) are shown. Experiments were repeated for 5 times. Statistical significance : * indicates $p<0.05$, and n.s. indicates $p >0.05$ for t-test.

(c) Number of LDs by *atg8*Δ deletion or Atg8 overexpression. The mean values of the ratio and standard errors (SE) are shown. Experiments were repeated for 3 times. Statistical significance : n.s. indicates $p >0.05$ for t-test.

Figure S5

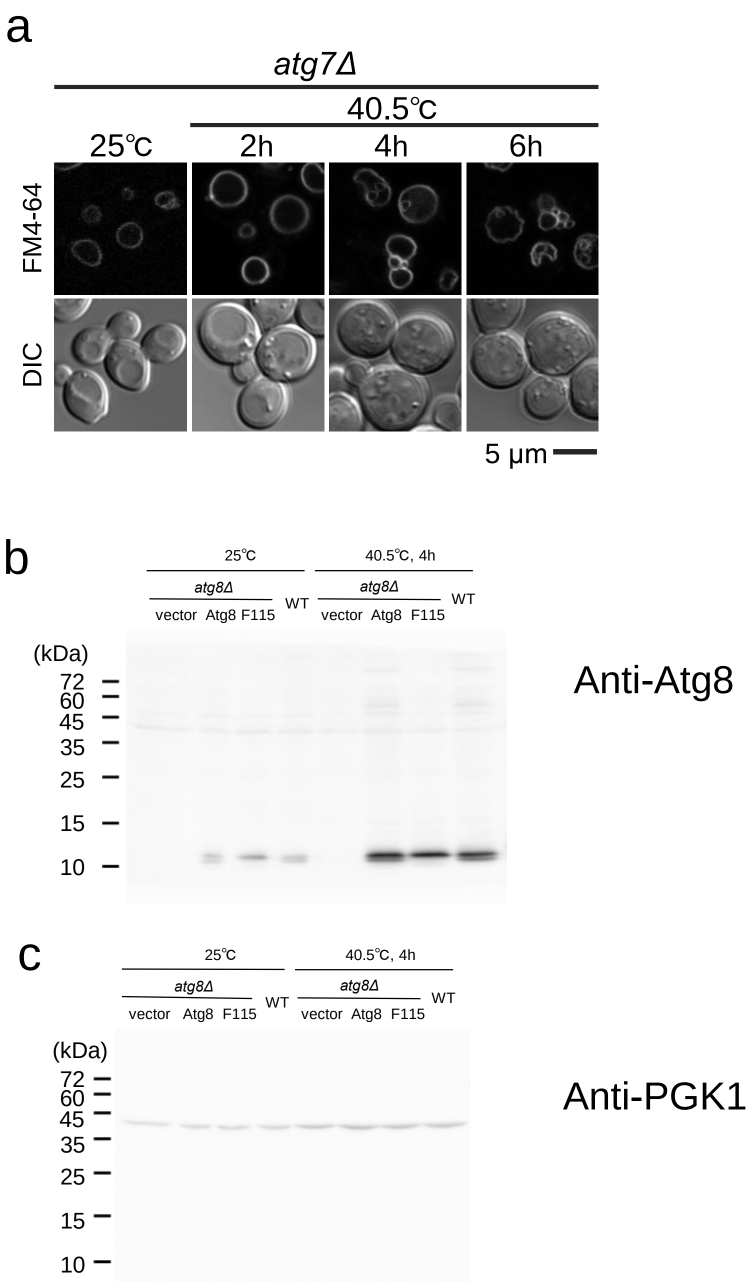

Figure S5. Lipidation-independent function of Atg8

(a) FM4-64 stained cells with equal degree of contrast adjustments of images between the cells grown at 25° C and heat stressed cells in Figure 3a.

(b) and (c) Uncropped images of the blots shown in Fig. 3d.

Figure S6

a

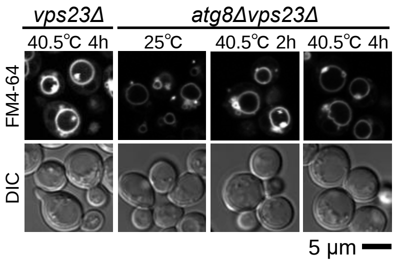

b

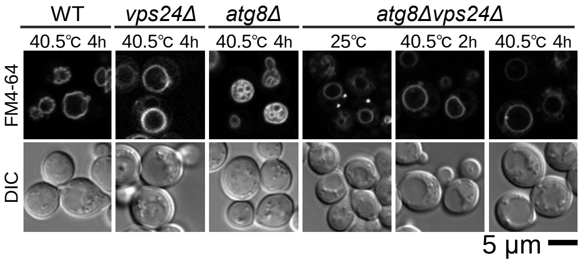

Figure S6. FM4-64 staining.

(a) FM4-64 staining of *vps23*Δ and *atg8*Δ*vps23*Δ cells. Scale bar, 5 µm.

(b) FM4-64-stained cells with the same image contrast adjustments between cells grown at 25° C and the heat-stressed cells in Fig. 4a.

Figure S7

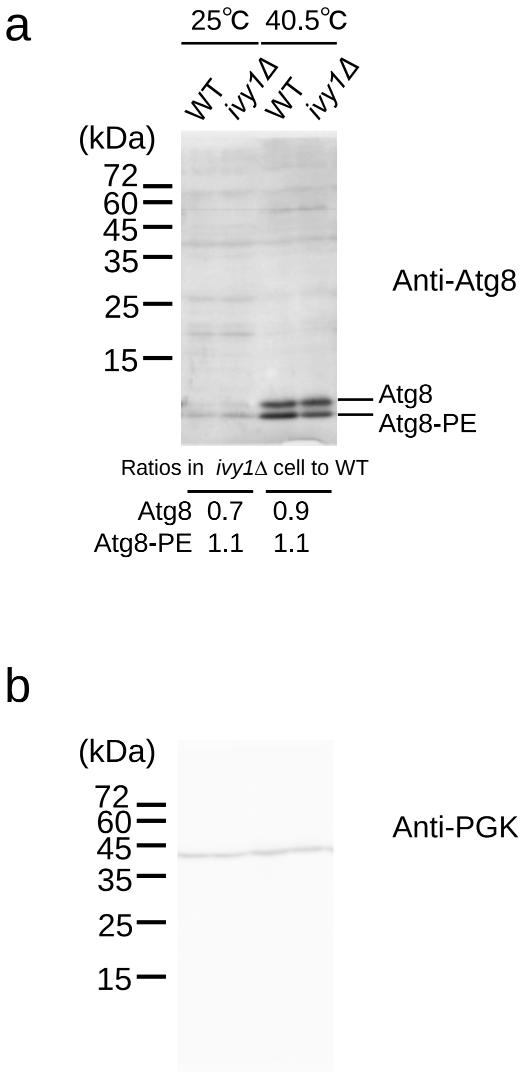

Figure S7. Atg8 expression in wild-type and *ivy1*Δ cells.

(a and b) Western blotting of anti-Atg8 and anti-PGK1 in wild-type and *ivy1*Δ cells at 25° C and 40.5° C for 6 h. Relative ratios of the level of Atg8 and Atg8-PE in *ivy1*Δ with respect to wild-type for each temperature are shown.

Figure S8

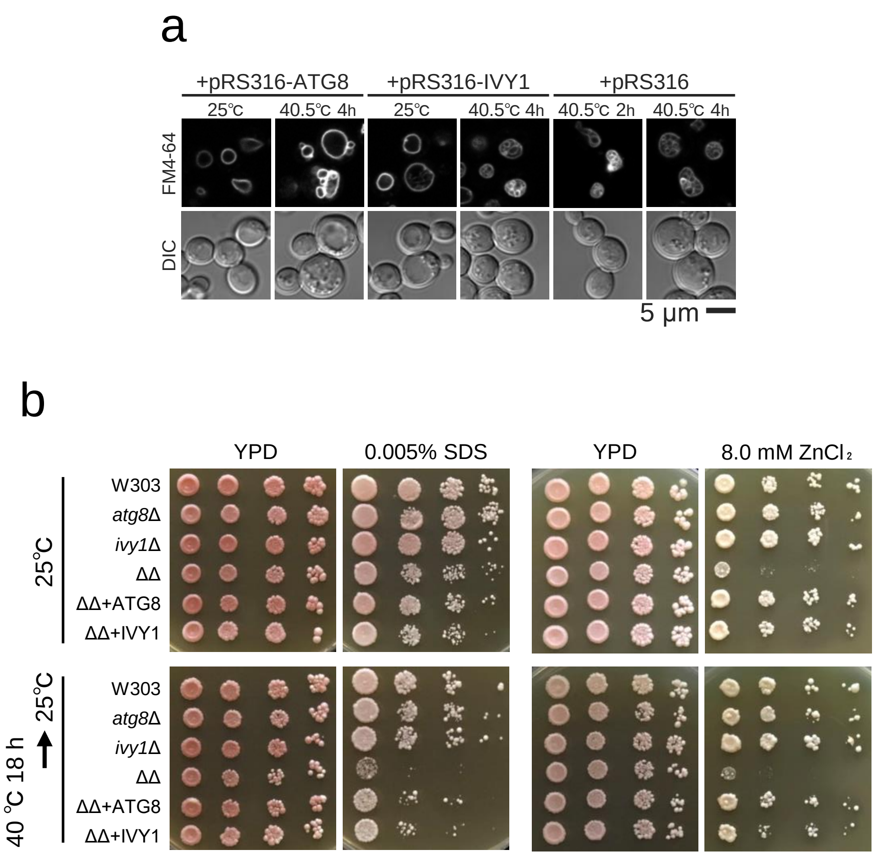

Figure S8. Suppression of invagination and growth sensitivities of *atg8Δivy1Δ* cells at 25° C by expression of either Atg8 or Ivy1.

(a) Vacuolar morphology. FM4-64 staining of *atg8Δivy1Δ* cells harboring a plasmid expressing Atg8 or Ivy1 or a vector (pRS316) are shown.

(b) Growth phenotype. Wild-type, *atg8Δ*, *ivy1Δ* *atg8Δivy1Δ* with a vector, *atg8Δivy1Δ* with a plasmid expressing Atg8, *atg8Δivy1Δ* with a plasmid expressing Ivy1 were diluted by ten-fold and spotted on YPD, YPD+0.005% SDS or YPD+8.0 mM $ZnCl_{2.}$ Cells were placed at 25° C or at 40° C for 18 h followed by the incubation at 25° C. Cells were incubated at 25° C for 3 days for YPAD, 4 days for YPD+ 0.005 % SDS, and 8 days for YPD +8.0 mM $ZnCl_2$ plates, respectively.

Table S1. Strains used in this study

Name	Genotype	Source/Reference
W303	MATa *ade2-1 can1-100 his3-12,16 leu2-3,112 trp1-1 ura3-1*	Rothstein
Y1373	W303a, *atg8Δ::KanMX*	This study
Y1408	W303α, *atg8Δ::KanMX*	This study
Y1390	W303a *PGK1-GFP::HIS3*	This study
Y1412	W303a *PGK1-GFP::HIS3, atg8*Δ::KanMX	This study
Y1444	W303a, *atg8Δ::KanMX*, *pRS306::URA3*	This study
Y1445	W303a, *atg8Δ::KanMX, pRS306-ATG8::URA3*	This study
Y1446	W303a, *atg8Δ::KanMX, pRS306-ATG8-F115::URA3*	This study
Y995	W303a, *vps23*Δ::KanMX	Ishii et al, 2018
Y1287	W303a, *vps24Δ::KanMX*	Ishii et al, 2018
Y1406	W303a, *atg8Δ::KanMX, vps24Δ::KanMX*	This study
Y1545	W303a, *atg8Δ::KanMX, vps 23Δ::KanMX*	This study
Y1375	W303a, *ivy1Δ::KanMX*	Ishii et al, 2018
Y1539	W303a, *atg8Δ::KanMX , ivy1Δ::KanMX*	This study
Y1542	W303a, *atg8Δ::KanMX , ivy1Δ::KanMX, PGK1-GFP::HIS3*	This study
Y1416	W303a, *atg7Δ::KanMX*	This study
BY4741	MATa *his3Δ1 leu2Δ0 ura3Δ0 met15Δ0*	Euroscarf
atg8	BY4741, *atg8Δ::KanMX*	Euroscarf
atg3	BY4741, *atg3*Δ::KanMX	Euroscarf
atg4	BY4741, *atg4*Δ::KanMX	Euroscarf
atg12	BY4741, *atg12*Δ::KanMX	Euroscarf
Y1410	W303, *IVY1-GFP::HIS3*	This study
Y1589	W303, *IVY1-GFP::HIS3, atg8*Δ::KanMX	This study
Y1590	W303, *IVY1-GFP::HIS3, vps24*Δ::KanMX	This study
BY20695	MATα, *his3-Δ1, leu2Δ0, met15&Delta0, ura3Δ0*	NBRP
Y1570	BY20695, *atg8*Δ::KanMX	This study
